# Neuroplasticity and tinnitus: the role of Brain-Derived Neurotrophic Factor in pathogenesis and treatment

**DOI:** 10.3389/fnins.2025.1620894

**Published:** 2025-07-08

**Authors:** Vincenzo Miranda, Sara Castiglioni, Jeanette A. Maier

**Affiliations:** ^1^Clinical Research Unit, GUNA S.p.a., Milan, Italy; ^2^Dipartimento di Scienze Biomediche e Cliniche, Università di Milano, Milan, Italy

**Keywords:** tinnitus, BDNF, inflammatory cytokines, synaptic plasticity, low-dose medicine

## Abstract

Subjective tinnitus is defined as the perception of sound in the absence of an external acoustic source, characterized by the phantom and persistent experience of noise or indistinct, internally generated non-verbal tones. The prevalence of chronic tinnitus is estimated to range from 5 to 42% of the global population. As a significant individual and societal issue, preclinical and clinical studies have been conducted to investigate this condition and explore potential therapeutic approaches. However, a comprehensive understanding of tinnitus and a definitive treatment remain elusive. In most cases, tinnitus arises from acquired and sustained hearing loss. However, the precise mechanisms and neuronal network models responsible for the perceptual generation and persistence of tinnitus are not yet fully understood. Animal studies have demonstrated that tinnitus is associated with synaptic plasticity dysfunction in multiple brain regions, including the auditory and limbic systems. Attention has been devoted to the contribution of inflammatory cytokines and deregulated levels of Brain-Derived Neurotrophic Factor (BDNF) to the pathogenesis of tinnitus. This narrative review aims to elucidate the functional structures and biological mechanisms underlying tinnitus and propose alternative novel therapeutic approaches.

## 1 Introduction

Tinnitus is the perception of sound in the absence of an external acoustic source, representing a phantom auditory experience without a corresponding acoustic signal or mechanism in the cochlea (Han et al., 2021; Messina et al., 2022). The prevalence of tinnitus varies across countries, largely due to differing definitions of the condition and challenges in measuring its severity (Han et al., 2021). A recent meta-analysis revealed that tinnitus affects 14% of adults globally, rising to 24% in older adults (Jarach et al., 2022). 10–60% of individuals with chronic tinnitus experience depressive disorders, and 28–45% exhibit significant symptoms of anxiety (Patil et al., 2023). Severe tinnitus is strongly associated also with insomnia, difficulty concentrating, reduced emotional and psychological wellbeing, and poor quality of life. In extreme cases, it can lead to functional disability and cognitive impairment (Blazer and Tucci, 2019). The unclear pathogenesis of tinnitus makes accurate diagnosis and effective treatment challenging. To date, no medications have been specifically approved by the U.S. Food and Drug Administration for the treatment of tinnitus (Kim et al., 2021).

**Figure 1:**
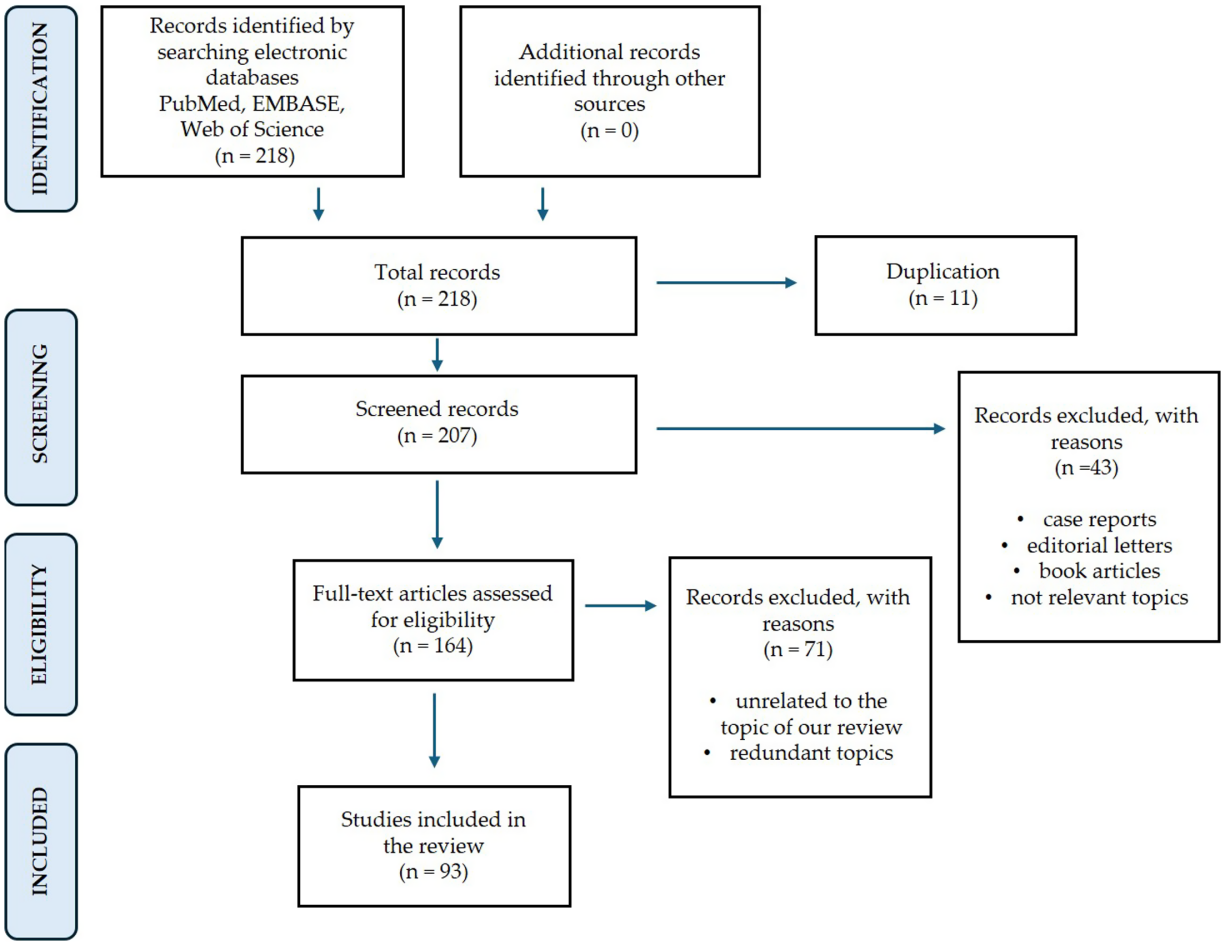
Diagram of the literature selection process. The search has been conducted from PubMed, EMBASE, and Web of Science. No relevant records were obtained from other sources. After exclusion of duplicates (11), 43 studies were excluded for the following reasons: editorial letters, book articles and not relevant topics. Therefore, 164 studies were eligible for full test screening. After exclusion of 71 studies for unrelated and redundant topics, a total of 93 studies were included in our study.

This narrative review provides a concise overview of the mechanisms underlying tinnitus and the key molecular players involved, with a particular emphasis on Brain-Derived Neurotrophic Factor (BDNF). We conducted a comprehensive review of studies across three electronic medical databases: PubMed, EMBASE, and Web of Science. The keywords used were “Tinnitus,” and “Central auditory system,” “Limbic System,” “Inflammatory cytokines,” “Neuroplasticity,” “BDNF,” “Low-Dose Medicine.” We included studies published in English with available abstracts and excluded case reports. We meticulously analyzed the full-text articles, selecting the most pertinent studies for inclusion in this review (Figure 1). The narrative review refers to papers searched up to February 22, 2025.

## 2 Understanding tinnitus: emerging insights into etiopathogenesis

**Figure 2:**
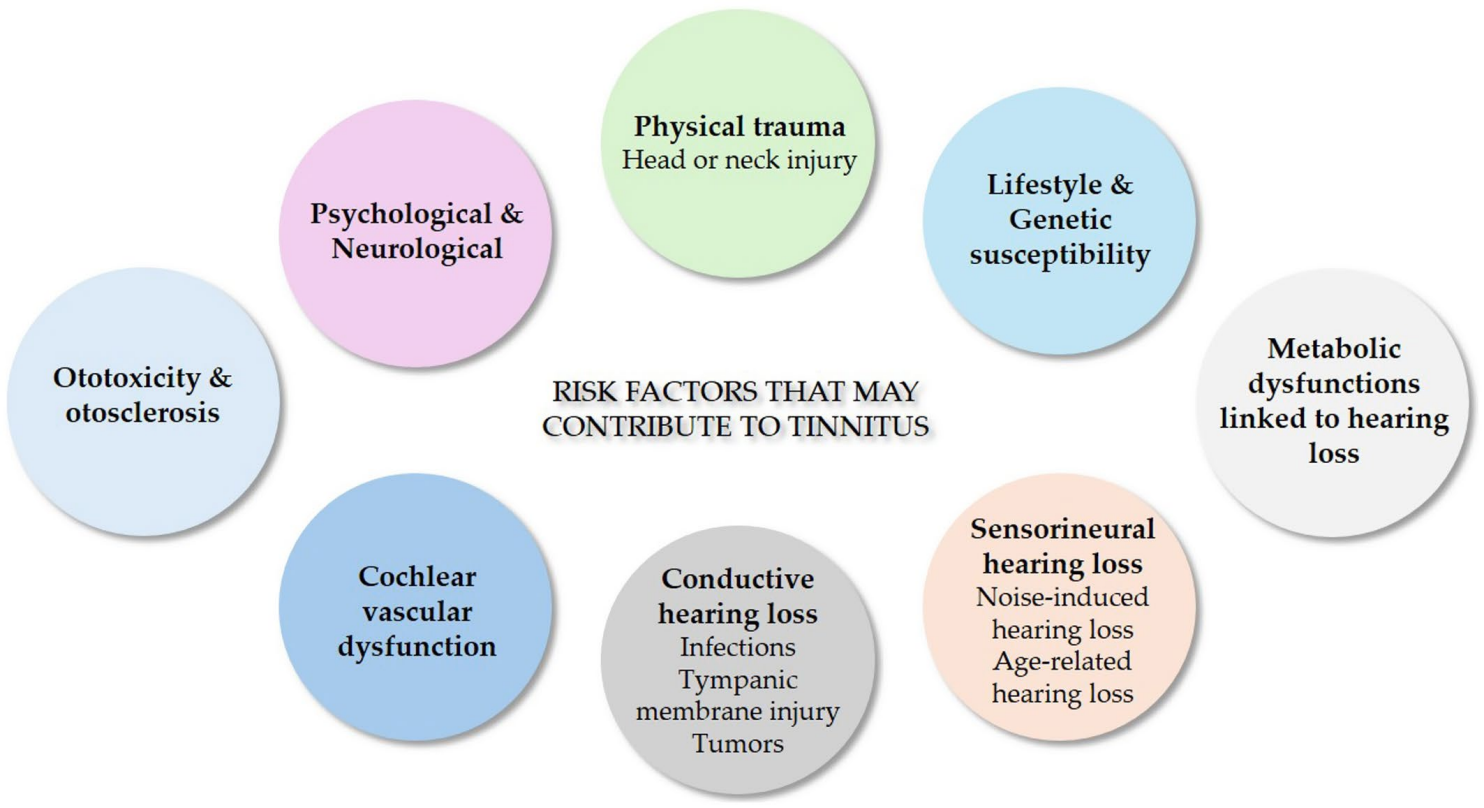
Risk factors contributing to tinnitus. Tinnitus induction is heterogeneous in nature as the onset can occur due to psychological and neurological issues, physical trauma, lifestyle and genetic susceptibility, metabolic dysfunctions, sensorineural hearing loss, conductive hearing loss, cochlear vascular dysfunction, ototoxicity, and otosclerosis.

The precise etiopathogenesis of tinnitus remains incompletely understood, with highly diverse underlying causes, as summarized in Figure 2. Tinnitus frequently occurs following damage to the auditory system, particularly due to exposure to excessive sound pressure levels. Evidence from both animal models and human studies has established a strong association between noise-induced hearing loss and tinnitus development (Henton and Tzounopoulos, 2021). Indeed, cochlear hair cells injured by loud sounds as well as by aging convert the aberrant sound vibrations into abnormal electrical signals, which the brain misinterprets as sound, leading to the perception of tinnitus (Fettiplace, 2017). This highlights the necessity of addressing hearing loss as a key factor in tinnitus prevention and management. Also, metabolic dysfunctions can lead to hearing loss and contribute to the generation or exacerbation of tinnitus through various pathways, including microvascular damage, neuropathy, direct effects on cellular metabolism within the inner ear, and systemic inflammation. Recently, hypertriglyceridemia and high total cholesterol/high-density lipoprotein cholesterol ratio were associated with increased risk of tinnitus in a Korean population (Lee et al., 2024). In addition, diabetes is strongly associated with an increased risk of tinnitus (Mousavi et al., 2021) through various mechanisms among which vascular damage, nerve damage, oxidative stress and low-grade systemic inflammation. Of interest, functional magnetic resonance imaging in type 2 diabetic individuals underscored a relation between the levels of Hemoglobin A1c, used to monitor glucose control, and decreased cerebral blood flow patterns in the right medial prefrontal gyrus, a region that is implicated not only in the auditory perception of tinnitus but also in how individuals experience and cope with it emotionally and cognitively (Xia et al., 2021).

Other contributors to tinnitus include environmental toxins, ototoxic medications (e.g., aminoglycosides, cisplatin, ethacrynic acid, furosemide, and salicylates) (Campo et al., 2013), and pathological conditions such as Ménière’s disease (Yoshida et al., 2011) and vestibular schwannoma (King et al., 2024). Risk factors for tinnitus extend beyond direct damage to the auditory system to include head trauma, ear infections, lifestyle habits, psychological problems, affective and psycho-emotional disorders, multiple physical or mental health conditions, and aging (Fagelson, 2022). Tinnitus may also be triggered by adverse life events such as grief, divorce, job loss, unemployment, unwanted retirement, and emotional stress (Han et al., 2021). Among these, post-traumatic stress disorder is particularly notable, affecting hundreds of millions globally, including approximately 1.45 billion people who survived conflicts between 1989 and 2015 (Patil et al., 2023).

Questions have arisen regarding a possible correlation between COVID-19 vaccination and the development of tinnitus (Ahmed et al., 2022). A systematic review by Arabzadeh Bahri et al. (2024) reported that patients who received COVID-19 vaccines occasionally experienced sensory disturbances, including smell or taste disorders, aural fullness, tinnitus, and headaches following the first or second vaccine dose. Conversely, a retrospective study by Aldè et al. (2023) demonstrated the efficacy of the COVID-19 vaccine in reducing vestibular and auditory disorders, including tinnitus, in a pediatric population with normal hearing or unilateral hearing loss. Among vaccinated children, the incidence of tinnitus was 1.4%, with symptoms resolving spontaneously within 24 h. In contrast, children who contracted SARS-CoV-2 showed a higher tinnitus incidence of 8.3%, with symptoms persisting for up to 5 days.

While no single “tinnitus gene” has been conclusively identified, evidence suggests that certain genetic variations may contribute to its development. Genetic predisposition appears to play a role in tinnitus severity, with specific genes linked to increased susceptibility (Amanat et al., 2020). Notably, a potential association has been identified between tinnitus and the BDNF Val66Met polymorphism, which introduces a functional missense mutation that impairs BDNF secretion from synapses (Coskunoglu et al., 2017; Yuksel et al., 2023). The BDNF gene is regulated by the BDNF antisense RNA gene (BDNF-AS), located downstream of BDNF. Polymorphisms in BDNF-AS are known to influence the auditory pathway and may increase the risk of chronic tinnitus (Yuksel et al., 2023). As a result, BDNF and BDNF-AS are emerging as candidate genes with the potential to significantly impact auditory performance.

## 3 Tinnitus: a multifaceted neurological phenomenon

**Figure 3:**
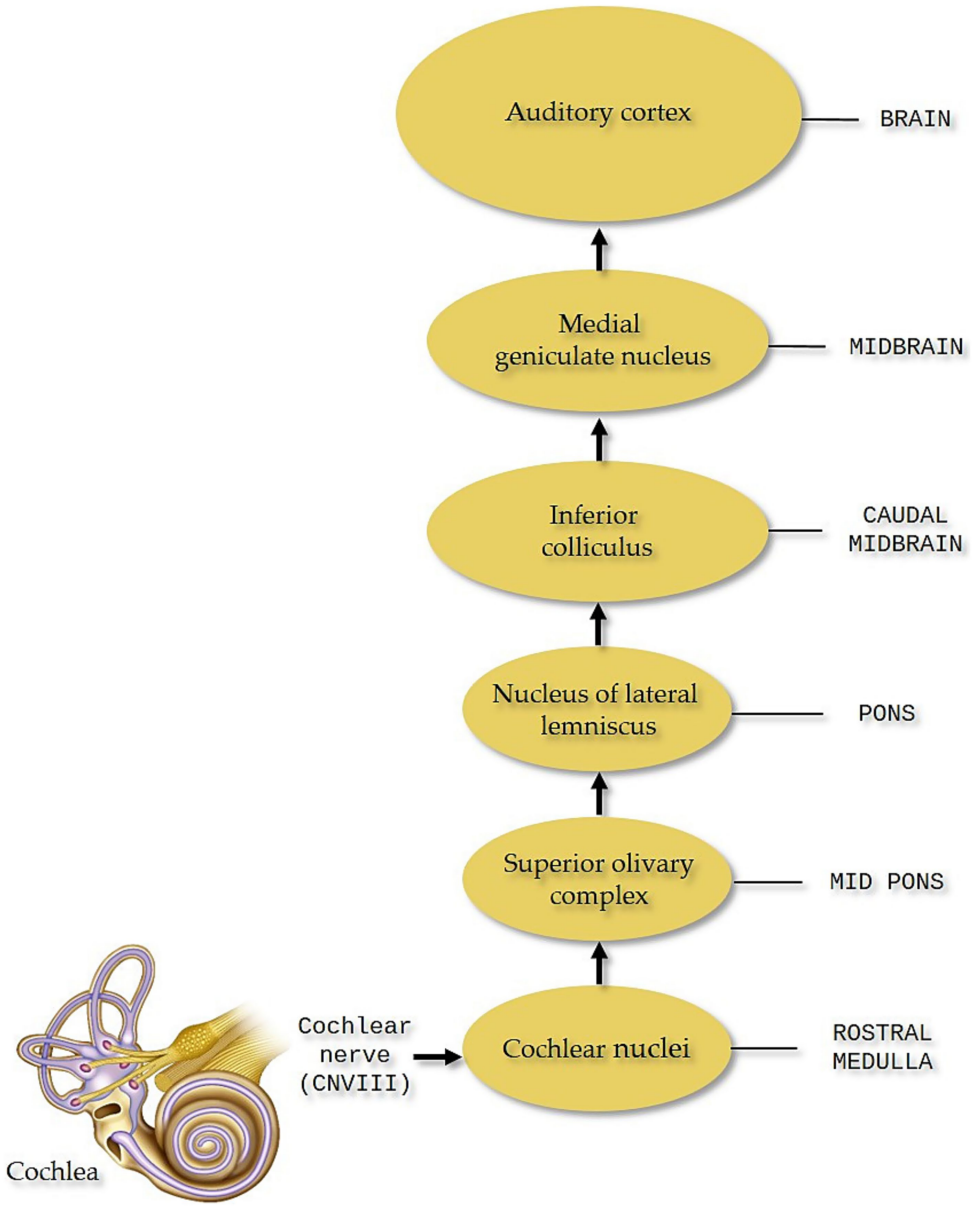
Central auditory system. The central auditory system originates from the cochlear nerve, which transmits auditory information from the cochlea to higher-order auditory structures in the brain for processing.

The initial theory regarding the origin of tinnitus suggested a cochlear basis. However, this perspective has evolved over time, with current understanding pointing to the involvement of not only the peripheral but also the central auditory system (Figure 3), and beyond to involve areas integrating auditory perception with emotional, attentional, and memory processes.

Many researchers support the hypothesis that cochlear damage triggers tinnitus by inducing abnormal hyperactivity in key areas, including the dorsal cochlear nucleus, thought to be a tinnitus-generating site, the inferior colliculus (IC), which integrates auditory pathway signals, the medial geniculate nucleus in the thalamus, a crucial hub for processing auditory signals, and the auditory cortex (Xu et al., 2019). This process is accompanied by reorganization of the central auditory system’s tonotopic map, disrupting the balance between excitatory and inhibitory neurotransmission across associated networks. Further research has demonstrated that tinnitus also engages non auditory brain areas, particularly those associated with attention, memory, emotional regulation, and cognitive processing such as the limbic system (Henton and Tzounopoulos, 2021; Singh et al., 2023; Xu et al., 2019; Davis and Johnsrude, 2003; Watanabe et al., 2008), the prefrontal cortex, the parietal lobes, the Default Mode Network (DMN) (Smitha et al., 2017; Shao et al., 2019) and the Dorsal Attention Network (DAN) (Rosemann and Rauschecker, 2023).

Hints about the involvement of some of these areas derive from clinical data. Notably, clinical cases of tinnitus cessation following cerebrovascular trauma affecting the caudate nucleus and putamen (Lowry et al., 2004) suggest the potential of striatal neuromodulation in tinnitus treatment. Abnormal excitability in the nucleus accumbens has been associated with emotional responses to sound and the severity of tinnitus and hyperacusis (Mahoney et al., 2011; Asokan et al., 2018). The cingulum cortex, integral to emotional reactivity, processing, and inhibitory control (Fan et al., 2024), has been linked to discomfort experienced by tinnitus patients (Singh et al., 2023). In tinnitus, disrupted functional connectivity within the auditory cortex, thalamic, limbic, and prefrontal regions, and networks like the DMN and DAN, has been observed (Rosemann and Rauschecker, 2023). Studies reveal reduced connectivity between DMN regions, such as the precuneus and superior parietal lobe, in patients with bothersome tinnitus (Schmidt et al., 2017). Conversely, long-term tinnitus has been associated with increased connectivity within the DAN.

Animal models of tinnitus are commonly induced using salicylates or prolonged exposure to high-intensity noise, both of which primarily affect outer hair cells by reducing their electromotility and leading to hyperactivity in the auditory cortex (Henton and Tzounopoulos, 2021). Dynamic imaging of cerebral microvascularization has identified the anterior cingulate cortex as a critical region involved in salicylate-induced tinnitus (Fan et al., 2024). Animal studies have demonstrated that tinnitus is closely associated with alterations in the synaptic plasticity of the limbic areas, particularly the hippocampus. This altered plasticity, which refers to the brain’s ability to strengthen or weaken connections between neurons, may impair the auditory gating mechanism.

**Figure 4:**
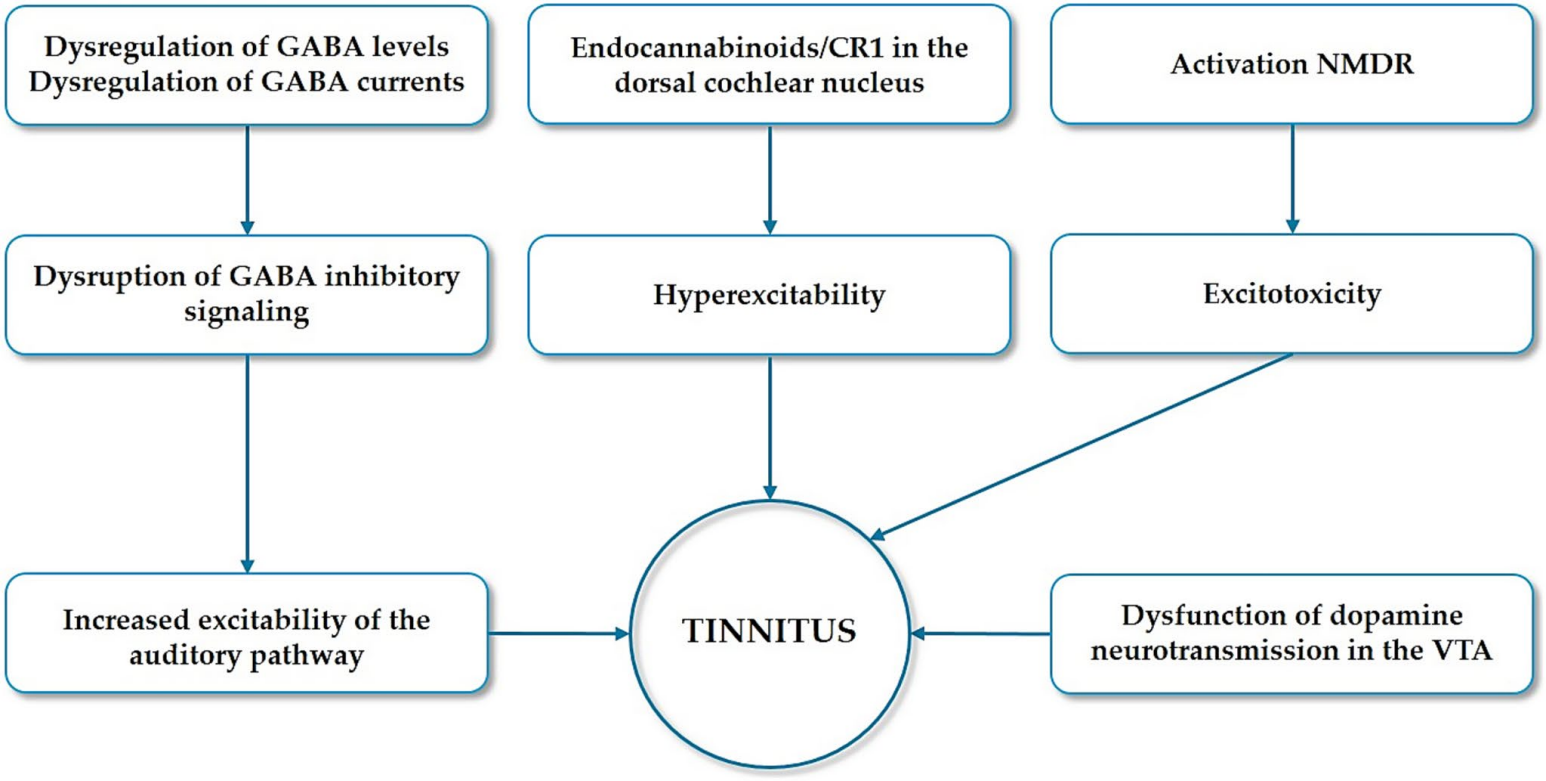
Dysregulation of neurotransmitter systems in the auditory pathway leading to tinnitus. Impaired GABAergic transmission, including altered GABA levels and inhibitory currents, leads to increased neuronal excitability in the auditory pathway. NMDA receptor dysfunction enhances glutamatergic activity and synaptic plasticity, while disruption of endocannabinoid signaling reduces inhibitory control over both GABA and glutamate release. Dopaminergic neurons in the ventral tegmental area (VTA) send projections to limbic structures, where dopamine modulates the processing of auditory signals. Together, these alterations contribute to a hyperexcitable auditory network, which underlies aberrant auditory processing.

Dysregulated neuroplasticity is mediated by the imbalance between various neurotransmitters. We rapidly overview the role of excitatory/inhibitory balance (glutamate vs. GABA) and neuromodulators like dopamine and endocannabinoids in tinnitus. In rats with noise-induced tinnitus, whole-cell patch-clamp recordings show that prolonged exposure to high-intensity sound does not change glutamatergic excitatory transmission while increasing the amplitude of inhibitory currents mediated by Gamma-Amino Butyric Acid (GABA), an inhibitory neurotransmitter which is present throughout multiple levels of the auditory pathway (Cunha et al., 2019). In the same experimental model, magnetic resonance spectroscopy (Brozoski et al., 2012; Isler et al., 2022) reveals decreased GABA levels in the medial geniculate body and systemic treatment with GABA agonists effectively suppresses behavioral evidence of tinnitus (Brozoski et al., 2007). These studies underscore the relevance of integrating various technical approaches to investigate various phenomena in the brains. In individuals with tinnitus, a human MRS study identified a deficiency of GABA in the right auditory cortex compared to healthy controls, and indicate that loud sounds may disrupt inhibitory transmission with consequent increased excitability of the auditory pathway (Sedley et al., 2015) (Figure 4).

Glutamate is the most common excitatory neurotransmitter in the auditory system and N-methyl-d-aspartate receptors (NMDARs) are the most widely distributed glutamate receptors in the auditory system. In animal models of tinnitus NMDARs are overactivated and tinnitus is relieved by NMDAR antagonists (Ruel et al., 2008; Ralli et al., 2014) (Figure 4). Surprisingly, reduced Glutamate concentration in the right auditory cortex was measured in humans with tinnitus without clinical relevant hearing loss (Isler et al., 2022). Clinical trials with NMDAR antagonists to treat tinnitus yielded contrasting results (Figueiredo et al., 2008; Suckfüll et al., 2011), probably because tinnitus is multifactorial and heterogeneous. We anticipate that future studies should stratify patients based on etiology to assess the pharmacological efficacy of NMDAR antagonists for tinnitus.

Endocannabinoid signaling also plays a critical role in modulating synaptic plasticity (Viveros et al., 2007). CB1 receptors, which are highly expressed in brain regions such as the auditory cortex, neocortex, hippocampus, basal ganglia, cerebellum, and brainstem, influence auditory function by modulating glutamatergic, glycinergic, and cholinergic neurotransmission (Bhat et al., 2023). In an experimental model of salicylate-induced tinnitus, CB1 receptors contribute to enhanced excitation in the dorsal cochlear nucleus circuitry (Smith and Zheng, 2016) (Figure 4). Research on the link between tinnitus and cannabinoid use in humans has yielded inconsistent results (Perin et al., 2020).

Dopamine, a key neurotransmitter in the basal ganglia, has been implicated in tinnitus development by modulating auditory pathway neurotransmission between the limbic system and the cochlea (Lopez-Gonzalez and Esteban-Ortega, 2005). Dopaminergic neurons in the ventral tegmental area project to the limbic structures, where dopamine influences auditory signal processing. Notably, injecting dopaminergic agonists into the nucleus accumbens has been shown to significantly reduce auditory gating in the hippocampus, suggesting that dopaminergic neurotransmission within the limbic system may contribute to tinnitus perception at the conscious level (Singh et al., 2023).

### 3.1 Tinnitus: the role of inflammatory cytokines

A systematic review of nine animal and 20 human studies reported changes in the primary and secondary auditory cortices, where increases in Tumor Necrosis Factor (TNF)-α and Interleukin (IL)-1β are accompanied by reductions in Interferon (IFN)-γ and astrocytic microglial activation, while IL-6 levels remain unchanged (Mennink et al., 2022). Tinnitus inducing noise triggers inflammasome activation, particularly NLRP3, within hours and sustained IL-18 elevation up to 10 days after noise exposure (Wang et al., 2019). In rodents, increased levels of TNF-α and IL-1β in the cochlea and dorsal cochlear nucleus, accompanied by astrocytic activation, were described (Mennink et al., 2022). Elevated levels of pro-inflammatory cytokines such as TNF-α and IL-1β have also been observed in the IC and medial geniculate body (MGB), a structure that integrates auditory inputs from the IC, reticular nucleus, and limbic structures while projecting to the auditory cortex, and TNF-α knock-out mice do not develop noise-induced tinnitus (Henton and Tzounopoulos, 2021). Of note, TNF-α and IL-1β directly affect synaptic plasticity along the auditory pathway. TNF-α induces endocytosis of GABA-A receptors, reducing inhibitory synapses and enhancing excitatory neurotransmission (Pribiag and Stellwagen, 2013), and IL-1β suppresses GABAergic currents (Wang et al., 2000). Severe tinnitus generates a vicious cycle because, as a source of stress, it activates the Hypothalamus-Pituitary–Adrenal axis, leading to increased levels of cortisol, reduced BDNF, and elevated norepinephrine, serotonin, and 5-hydroxyindoleacetic acid (Patil et al., 2023). Cortisol release may exacerbate the activity of the glutamate receptors NMDA and Amino-Methyl Phosphonic Acid (AMPA), contributing to worsen tinnitus (Patil et al., 2023).

Clinical studies have yielded mixed results regarding cytokine levels in individuals with tinnitus. Weber et al. reported significantly elevated IL-6 levels in tinnitus patients (Weber et al., 2002), while Heider et al. found no changes in IL-6 but observed reduced IL-10 levels (Haider et al., 2020). Interestingly, IL-10 levels increased with the chronicization of tinnitus, while levels of IL-1α, IL-1β, IL-2, IFN-γ, TNF-α, and Transforming Growth Factor (TGF)-β remained unchanged (Weber et al., 2002; Haider et al., 2020). Discrepancies between animal and human studies could arise from differences in measurement timing relative to tinnitus onset. Animal studies often measure TNF-α immediately after tinnitus induction, while clinical studies assess cytokines in chronic tinnitus patients, potentially after TNF-α levels have normalized. Moreover, human studies measure cytokine levels in blood, which may not reflect localized changes in auditory structures.

Mediators released by platelets appear to play a role in tinnitus onset. Notably, increased mean platelet volume and platelet distribution width have been observed in tinnitus patients (Yazici and Cihan, 2023) and may result in impaired perfusion of the inner ear and tinnitus (Kemal et al., 2016). Moreover, larger platelets are metabolically and enzymatically more active, contain more granules and produce higher levels of biologically active factors, including vasoactive and thrombotic molecules (Bekler et al., 2015). Activated platelets release cytokines like IL-6, IL-8, and TNF-α within minutes, potentially contributing to prothrombotic conditions or thrombotic events in the internal auditory artery, leading to cochlear hypoperfusion and tinnitus. Additionally, platelets, which have glutamate transporters, remove glutamate from the bloodstream and a reduced glutamate uptake, as reported in aging, may exacerbate tinnitus by increasing excitotoxicity.

### 3.2 Tinnitus: the role of BDNF

BDNF (Brain-Derived Neurotrophic Factor) plays a crucial role in maintaining a healthy brain. It is essential for neurogenesis, synaptic plasticity, and neuronal survival. These functions are vital for cognitive abilities, emotional regulation, and even resilience to stress. Given its importance, when BDNF function is disrupted, it can lead to cognitive impairment and is implicated in various mental illnesses, including depression (Numakawa and Kajihara, 2025).

As mentioned above, genetic studies suggest that dysregulated BDNF may play a role in the development of tinnitus. BDNF, the most prevalent neurotrophin in mammals’ brain, is widely expressed in the central and peripheral nervous systems. It plays a crucial role in various biological processes (Table 1), including neuronal survival, growth, differentiation, and synaptic plasticity also in auditory system. BDNF supports the survival and growth of afferent fibers that connect the vestibular organ to the cochlear regions responsible for mapping low-frequency sound information to central auditory nuclei and higher auditory centers (Singer et al., 2014).

**Table 1:** Main activities of Brain-Derived Neurotrophic Factor.

Brain-Derived Neurotrophic Factor activity	
Neuronal survival	Promotes survival of existing neurons and prevents cell death.
Neurogenesis	Stimulates the growth and differentiation of new neurons from neural stem cells.
Axonal and dendritic growth	Enhances outgrowth and branching of axons and dendrites, potentiating connectivity.
Synaptogenesis and synaptic stability	Stimulates generation of new synapses and enhances synaptic strength and stability.
Synaptic plasticity	Improves synaptic plasticity, essential for learning and memory.
Anti-inflammatory activity	Modulates release of proinflammatory cytokines.
Anti-Reactive Oxygen Species (ROS) activity	Counteracts ROS production and maintains redox homeostasis.
Anti-degenerative and anti-aging activity	Reduces Tau phosphorylation and promotes β-Amyloid protein uptake and degradation thus supporting brain health during aging.
Modulation dopaminergic, serotoninergic, cholinergic and GABAergic signaling	Modulates dopaminergic, serotoninergic, cholinergic circuits and GABAergic signaling.
Development of neuroprotective mechanisms.	Restores long-term-potentiation and activates CREB leading to increased expression of genes encoding proteins involved in neuroprotection.

BDNF is initially synthesized as a precursor protein, proBDNF, which is cleaved into its mature form (mBDNF). These two forms exert distinct and sometimes opposing effects on neuronal function. mBDNF primarily signals through the tropomyosin-related kinase B (TrkB) receptor, triggering pathways involved in neuronal survival, differentiation, and synaptic plasticity (Brigadski and Leßmann, 2020). In contrast, proBDNF binds to the p75 neurotrophin receptor (p75NTR), typically promoting neuronal apoptosis, synaptic pruning, and neurodevelopmental remodeling (Ali et al., 2024). During early development, proBDNF plays a key role in neuro-glial interactions, synaptogenesis, apoptosis regulation, and the elimination of improperly formed connections. In adulthood, mBDNF contributes to neurotransmission efficiency by promoting neuritic growth, dendritic arborization, dendritic spine development, and the downregulation of GABA receptor expression, reducing GABAergic interneuron excitability. Additionally, mBDNF enhances neuroprotection by mitigating glutamatergic excitotoxicity, reducing Ca^2+^ influx, and activating cAMP response element-binding protein (CREB), which promotes the expression of neuroprotective genes. Furthermore, BDNF has been shown to prevent cellular damage and neuronal loss following permanent sensorineural hearing impairment (Schimmang et al., 2003; Barclay et al., 2011; Wong and Ryan, 2015).

As a key modulator of neuroplasticity, BDNF counteracts the effects of pro-inflammatory cytokines (Charlton et al., 2023), which are critical mediators of neurodegeneration. BDNF also serves as a biomarker of stress-related responses; acute stress elevates BDNF levels (Linz et al., 2019), whereas chronic stress leads to BDNF downregulation (Robinson et al., 2021). Cortisol appears to regulate BDNF expression (Issa et al., 2010), with its daily fluctuation mirroring the cortisol circadian rhythm—peaking at approximately 8:00 a.m. and gradually decreasing throughout the day (Ehrhardt et al., 2024). Notably, physical exercise enhances BDNF release in the brain (Sleiman et al., 2016).

In healthy individuals, average plasma BDNF levels are approximately 92.5 pg/mL (range: 8.0–927.0 pg/mL), with higher concentrations observed in women. However, BDNF levels decline with advancing age and increased body weight in both sexes (Lommatzsch et al., 2005; Roh et al., 2022). Reduced circulating BDNF is associated with the onset of several neurodegenerative diseases, including Alzheimer’s, Parkinson’s, Multiple Sclerosis, and Huntington’s disease (Ibrahim et al., 2022), as well as psychiatric disorders such as major depressive disorder (Wang et al., 2011; Mojtabavi et al., 2020). Also, tinnitus patients exhibit decreased serum and hair levels of BDNF (Coskunoglu et al., 2017; Ranjbar et al., 2023), suggesting a potential link between tinnitus pathophysiology and BDNF signaling. Furthermore, specific single nucleotide polymorphisms (SNPs) in the BDNF gene, including rs6265 (Val66Met), rs2030324, and rs1491850, have been associated with altered BDNF serum levels and brainstem auditory evoked response (BAER) test results (Yuksel et al., 2023).

**Figure 5:**
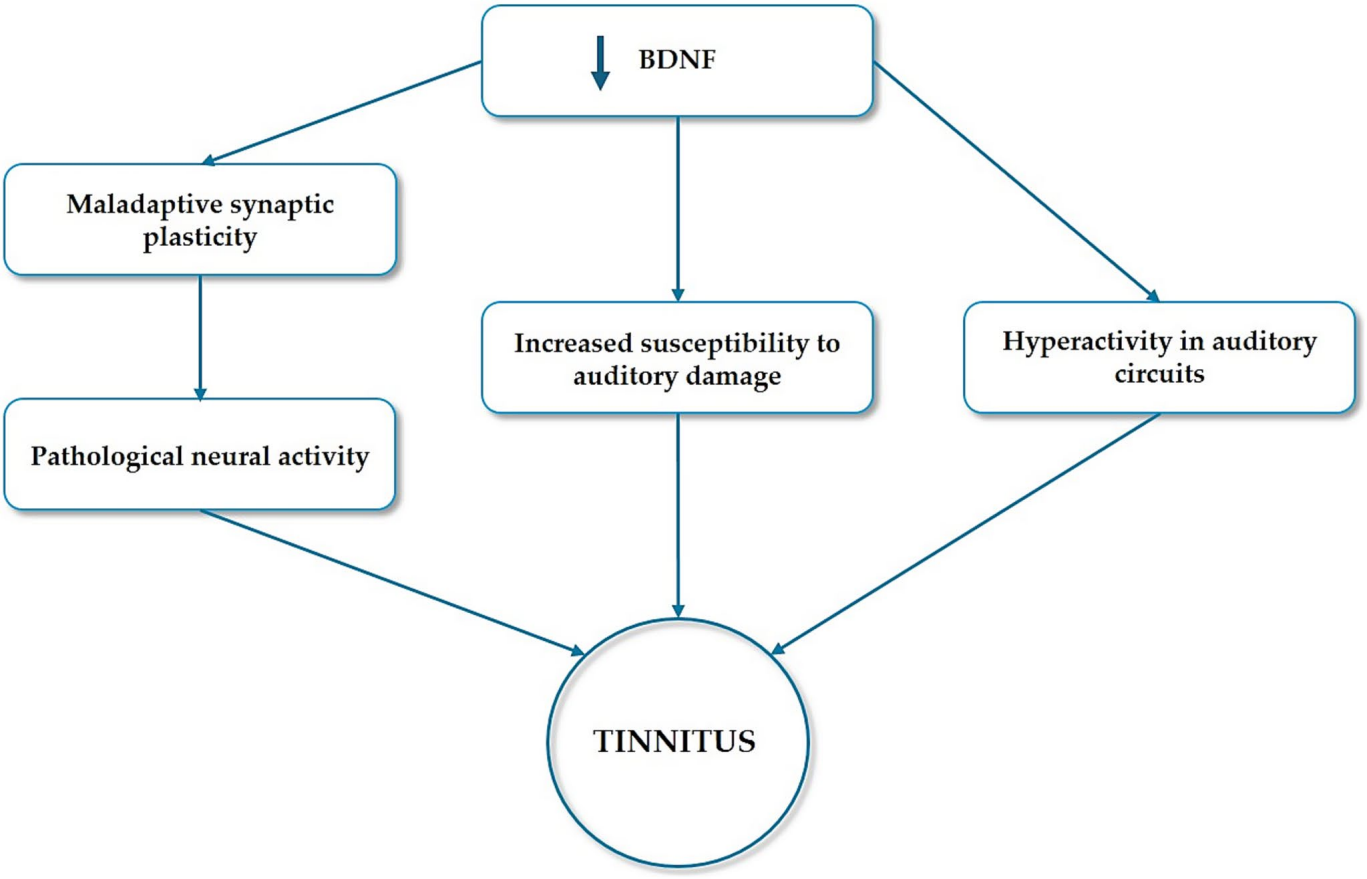
The role of BDNF dysregulation in tinnitus pathophysiology. Tinnitus is believed to result from maladaptive synaptic plasticity in the auditory and limbic systems. As a key regulator of synaptic plasticity, BDNF dysregulation may contribute to the pathological neural activity underlying tinnitus. Moreover, BDNF supports the survival and function of neurons in the auditory pathway. Reduced levels of BDNF have been implicated in increased susceptibility to auditory damage and the subsequent development of tinnitus. Additionally, tinnitus is often associated with hearing loss, during which BDNF-mediated compensatory mechanisms in the auditory system may lead to hyperactivity in auditory circuits, a hallmark of tinnitus.

Experimental studies indicate that changes in BDNF expression influence tinnitus-like behavior. Noise-induced hearing loss, a common precursor to tinnitus, is associated with altered BDNF levels in the auditory cortex and other brain regions. Moreover, the limbic system, which plays a crucial role in the emotional and cognitive aspects of tinnitus, is particularly susceptible to BDNF dysregulation (Camuso et al., 2022). Altered BDNF signaling in limbic structures such as the hippocampus and amygdala may contribute to the heightened distress often experienced by individuals with tinnitus. The role of reduced levels of BDNF in the pathophysiology of tinnitus are summarized in Figure 5.

## 4 BDNF as a potential therapeutic strategy for tinnitus

The regulation of BDNF levels presents a promising avenue for tinnitus treatment. Notably, physical exercise and specific auditory training protocols have been shown to elevate BDNF levels, potentially mitigating tinnitus severity (Carpenter-Thompson et al., 2015). However, effective delivery of BDNF to targeted brain regions remains a significant challenge due to its short half-life. Interestingly, circulating BDNF is capable of crossing the Blood–Brain Barrier (BBB) via a rapid, saturable transport system (Pan et al., 1998). This efficient unidirectional transport suggests that peripheral administration of BDNF could facilitate its entry into the Central Nervous System (CNS), thereby promoting neuronal regeneration and repair.

In recent years, the administration of exogenous BDNF has been explored as a therapeutic strategy for neurodegenerative diseases. Both human and animal studies have demonstrated that BDNF supplementation is associated with improved cognitive function and reduced synaptic dysfunction. In developing neural circuits, exogenous BDNF has been shown to enhance dendritic growth, thereby increasing the complexity of pyramidal neurons, particularly in the visual cortex (Bathina and Das, 2015). Additionally, exogenous BDNF administration has been found to prevent and mitigate neurodegenerative changes associated with brain aging, restoring long-term potentiation and spatial memory in aged animal models (Nagahara et al., 2009).

Despite its therapeutic potential, the administration of exogenous BDNF presents several challenges (Table 2), primarily related to its bioavailability in the brain. If the delivered concentration is insufficient, it may fail to induce the desired neuroprotective effects. Conversely, excessive BDNF levels could have paradoxical consequences, including the downregulation of TrkB receptors and a subsequent reduction in intracellular signaling pathways crucial for neuronal function and plasticity. These limitations underscore the need for novel strategies to optimize BDNF delivery and regulation, potentially enhancing its therapeutic utility for tinnitus and other neurodegenerative conditions. In rodents and primates, oral administration of BDNF has yielded limited success due to its molecular properties, including its moderate size and charge, which hinder its transport across the intestinal barrier and BBB (Nagahara and Tuszynski, 2011). Previous studies have identified additional limitations associated with neurotrophin administration in humans, particularly concerning dosage and pharmacokinetics (Thoenen and Sendtner, 2002). A promising approach to achieving precise physiological regulation involves the use of low-dose substances, as supported by extensive literature (Gariboldi et al., 2009; Uberti et al., 2018). Low Dose Medicine (LDM) emerged from the intersection of molecular biology and psycho-neuro-endocrine-immunology, initially evolving from research in nanopharmacology (Fioranelli and Roccia, 2014; Bernasconi, 2018). The mechanism of action of low-dose cytokines, hormones, neuropeptides, and growth factors is based on the sensitization or activation of cellular and plasma receptors, driven by their high dilution. These molecules operate within a physiological range, from approximately 10^−6^ M (micrograms) for hormones to 10^−15^ M (femtograms) for other signaling molecules (Castiglioni et al., 2017; Cazzaniga et al., 2024). Nearly two decades of research on LDM has validated its conceptual foundation and demonstrated the efficacy and safety of therapeutic interventions using orally administered sub-nanomolar doses of signaling molecules, as exemplified by low-dose IL-2 therapy. Low-dose IL-2 therapy, a novel immunomodulatory strategy that re-establish immune homeostasis, is an emerging and promising treatment strategy for various autoimmune, inflammatory, and, increasingly, neurodegenerative diseases (He et al., 2020; Yuan et al., 2023; Zhang et al., 2024). This approach specifically targets and expands regulatory T cells (Matsuoka et al., 2013), thus offering a more precise therapeutic intervention with a promising efficacy and safety profile across a growing spectrum of immune-mediated and neurodegenerative disorders.

**Table 2:** Challenges and opportunities of BDNF-based therapy in tinnitus.

Aspect	Challenge	Possible solution
Delivery to CNS	Limited blood–brain barrier permeability of BDNF	Potential use of low-dose BDNF?
Pharmacokinetics	Short half-life of BDNF	Use of low-dose, high-frequency regimens to maintain stable levels
Dosage optimization	High doses may cause receptor desensitization or paradoxical effects	Potential use of low dose BDNF?
Heterogeneity of tinnitus	Multifactorial etiology complicates treatment response	Stratified clinical trials based on genetic, neurophysiological, and psychological profiles
Clinical evidence	Only preclinical data	Planned clinical trials approved by AIFA/EMA

Low-dose BDNF may represent a novel therapeutic avenue in tinnitus management. Recently, low-dose BDNF has been approved by the Agenzia Italiana del Farmaco-AIFA for therapeutic use, and clinical trials are planned in the near future. In an experimental model, the evidence that low-dose BDNF exhibits a high capacity to traverse the enterohepatic circulation and remains detectable in the bloodstream for at least 24 h (Molinari et al., 2020) suggests that it may represent a promising candidate for therapeutic supplementation. This study also shows that in the brain low-dose BDNF maintains redox homeostasis, limits the effects of pro-inflammatory cytokines, significantly increases astrocytic vitality, reduces Tau phosphorylation and promotes β-Amyloid protein uptake and degradation. Additionally, low-dose BDNF treatment for 24 h led to an upregulation of endogenous BDNF expression, indicating its potential to enhance endogenous BDNF production through stimulation and induction mechanisms. Randomized controlled clinical trials are necessary to validate the efficacy of low dose BDNF in restoring impaired functions and modulating neuroplasticity.

## 5 Conclusion

Tinnitus is characterized by dysfunctions in synaptic plasticity across multiple brain regions. Understanding this widespread neural involvement is crucial for developing effective therapeutic strategies. Given the central role of BDNF in regulating neuroplasticity, alterations in BDNF levels may contribute to the pathogenesis of tinnitus. Consequently, the administration of exogenous BDNF—already investigated in the context of neurodegenerative disorders—could represent a promising approach for tinnitus treatment. While challenges in its clinical translation remain, preliminary evidence supports the potential of low-dose BDNF therapy as a novel intervention. Rigorous, well-designed research focusing on mechanistic insights, optimized delivery, and stratified clinical trials is crucial to unlock the full therapeutic potential of low-dose BDNF for individuals suffering from this debilitating condition.
